# Retrospective analysis on confirmation rates for referred positive rotavirus samples in England, 2016 to 2017: implications for diagnosis and surveillance

**DOI:** 10.2807/1560-7917.ES.2020.25.43.1900375

**Published:** 2020-10-29

**Authors:** Cristina C Celma, Stuart Beard, Amy Douglas, Shan Wong, Nana-Kwame Osafo, Matthew Hannah, Ashleigh Hale, Gabrielle Huggins, Shamez Ladhani, Jake Dunning

**Affiliations:** 1 Enteric Virus Unit, National Infection Service Laboratories, Public Health England, London, United Kingdom; 2 These authors contributed equally to this work; 3 Gastrointestinal Pathogens Unit, National Infection Service Laboratories, Public Health England, London, United Kingdom; 4 High Containment Microbiology, National Infection Service Laboratories, Public Health England, London, United Kingdom; 5 Immunisations and Countermeasures, National Infection Service, Public Health England, London, United Kingdom

**Keywords:** Rotavirus, surveillance, diagnosis, screening, rapid test

## Abstract

**Background:**

Rapid diagnostic tests are commonly used by hospital laboratories in England to detect rotavirus (RV), and results are used to inform clinical management and support national surveillance of the infant rotavirus immunisation programme since 2013. In 2017, the Public Health England (PHE) national reference laboratory for enteric viruses observed that the presence of RV could not be confirmed by PCR in a proportion of RV-positive samples referred for confirmatory detection.

**Aim:**

We aimed to compare the positivity rate of detection methods used by hospital laboratories with the PHE confirmatory test rate.

**Methods:**

Rotavirus specimens testing positive at local hospital laboratories were re-tested at the PHE national reference laboratory using a PCR test. Confirmatory results were compared to original results from the PHE laboratory information management system.

**Results:**

Hospital laboratories screened 70.1% (2,608/3,721) of RV samples using immunochromatographic assay (IC) or rapid tests, 15.5% (578/3,721) using enzyme immunoassays (EIA) and 14.4% (535/3,721) using PCR. Overall, 1,011/3,721 (27.2%) locally RV-positive samples referred to PHE in 2016 and 2017 failed RV detection using the PHE reference laboratory PCR test. Confirmation rates were 66.9% (1,746/2,608) for the IC tests, 87.4% (505/578) for the EIA and 86.4% (465/535) for the PCR assays. Seasonal confirmation rate discrepancies were also evident for IC tests.

**Conclusions:**

This report highlights high false positive rates with the most commonly used RV screening tests and emphasises the importance of implementing verified confirmatory tests for RV detections. This has implications for clinical diagnosis and national surveillance.

## Introduction

Rotavirus (RV) infection is a common cause of severe watery diarrhoea in young individuals around the world [1]. In healthy individuals, the disease is usually mild and self-limiting, with symptoms lasting between 3 and 8 days. However, in very young or immunocompromised patients, RV infection can cause more severe manifestations including fever, vomiting, abdominal pain and dehydration. There is no specific treatment for RV infection; oral rehydration and intravenous fluid supplementation can be administrated to prevent or treat severe dehydration.

Prior to routine RV vaccination in the United Kingdom (UK), the burden of RV disease was estimated to be 750,000 diarrhoea episodes [2], and 14,300 diarrhoea-related hospital admissions of children under the age of five [3] every year, representing a considerable healthcare cost. In 2013, a monovalent live-attenuated RV vaccine was introduced into the UK infant immunisation programme as a two-dose schedule at 8 and 16 weeks of age. The programme resulted in a 77% reduction of laboratory-reported RV infections [4,5], and a 26% decrease in gastroenteritis-associated hospitalisation in young children [4,6]. It is estimated that the RV immunisation programme was associated with a GBP 12.5 million (EUR 13.7 million converted on 9 Sep 2020) saving in RV-associated healthcare costs within a year of implementation [6].

RV episodes are typically seasonal with most cases occurring during the winter and spring months (January to April in the temperate northern hemisphere). Following the introduction of RV immunisation, seasonal patterns have shifted, with shorter and more delayed periods of RV disease activity observed in some countries such as the United States (US) and Belgium [7-9].

In England, National Health Service (NHS) hospital laboratories routinely test stool samples from patients with gastroenteritis for a number of viruses including RV in order to confirm the diagnosis and inform clinical management. A variety of detection methods are used by NHS hospital laboratories for RV screening [10], such as immunoassay-based methods (i.e. enzyme-linked immunosorbent assay and immunochromatographic) and molecular assay-based methods (i.e. reverse transcription and PCR).

As part of national surveillance of the RV immunisation programme in England, NHS hospital laboratories are actively requested to submit all positive RV stool samples to the Public Health England (PHE) Virus Reference Department (VRD) Enteric Virus Unit (EVU) reference laboratory for confirmation and additional characterisation to support the molecular surveillance of circulating RV strains. In recent years, an increasing number of positive RV samples submitted by NHS hospital laboratories failed molecular characterisation. Therefore, a confirmatory PCR detection test was implemented at the PHE reference laboratory for all RV samples received; this PCR test is performed before attempting molecular characterisation. The PHE reference laboratory confirmatory PCR detection test identified a considerable proportion of RV-negative samples, suggesting a high rate of false positive results at some local NHS hospital laboratories.

The aim of this study was to determine the proportion of samples that tested positive for RV in local NHS hospital laboratories, were submitted to the PHE reference laboratory and were also positive using the PHE reference laboratory confirmatory PCR test. The study also aimed to compare the results of the PHE reference laboratory confirmatory PCR test with the results from the original RV testing method used by the NHS hospital laboratory. Variations in performance of the different tests during and outside the RV season were also assessed as part of the analysis.

## Methods

### Data source

Samples received at the PHE reference laboratory from 5 January 2016 to 28 December 2017 were included in the analysis. Data were extracted from the PHE reference laboratory information management system, which contains all the information provided in the laboratory request form, along with the results of the PHE reference laboratory confirmatory PCR test. For samples where the RV screening method was not stated on the laboratory request form, the individual laboratories were contacted directly.

### Segregation of tests and methodologies

Commercial rapid tests, enzyme immunoassays (EIA) and PCR tests were identified in this study. Of the seventeen types of commercial rapid tests used, six targeted RV only (RORT1 to RORT6) and 11 were designed for dual detection of RV and adenovirus (RART7 to RART17). For EIA, three subgroups were created: two commercially available kits (commercial EIA1 and commercial EIA2) and one group including other commercial or in-house tests (other EIAs). The PCR category was subdivided into four groups, including commercial (PCR1 to PCR3) and PCR assays developed in-house (in-house PCRs).

### Rotavirus detection–PHE reference laboratory confirmatory PCR test targeting VP6 gene

Patients’ stool samples received from referring laboratories across England were tested using real-time PCR (PHE reference laboratory confirmatory PCR) to determine the presence of RV RNA (n = 3,729). Partial amplification of the VP6 gene was performed as previously described [11,12] with modifications. Briefly, nucleic acid was extracted from 200μl of 10% faecal suspensions by automatic RNA extraction platform (MP96, Roche, Almere, the Netherlands or Qiasymphony, Qiagen, Hilden, Germany). A reverse transcription step was carried out with random hexamers (Invitrogen) followed by PCR amplification using primers VP6-F: 5’-GAC GGV GCR ACT ACA TGG T-3’; VP6-R: 5’-GTC CAA TTC ATN CCT GGT GG-3’; and probe VP6P: FAM5’-CCA CCR AAY ATG ACR CCA GCN GTA -3’ MGB. cDNA was initially heated at 50 °C for two minutes and 95 °C for 2 min, followed by 35 PCR cycles at 95 °C for 15 s and 60 °C for one minute. Mengovirus was used as an internal process control (added before the nucleic acid extraction step) and detected using primers MengoF: 5’-GCG GGT CCT GCC GAA AGT-3’, MengoR5’-GAA GTA ACA TAT AGA CAG ACG CAC AC-3’ and probe: MengoP5’-VIC-ATC ACA TTA CTG GCC GAA GC-MGB-3’.

The limit of detection of the PHE reference laboratory confirmatory PCR test was determined by 10-fold serial dilutions of in vitro transcribed single stranded RNA derived from simian rhesus rotavirus (RRV) segment S6 as template. Copy number was calculated using the formula:

copy number (molecules/µL) = [concentration (ng/µL) × 6.022 × 10^23^ (molecules/mol)]/ [length of amplicon × 650 (g/mol) × 10^9^ (ng/g)].

The limit of detection of 3.4 × 10^3^ copies of target RNA was determined as the lowest copy number that produced positive results in duplicates for two independent tests.

### Specific PCRs targeting NSP3 gene

A subset of 59 samples was identified for further testing. Criteria for selection were based on: (i) samples with a positive result by rapid tests but negative by PHE reference laboratory confirmatory PCR; (ii) samples received in the current (at the time) RV season to mitigate sample degradation. A specific PCR targeting NSP3 gene was performed as described elsewhere [13]. Briefly, after the reverse transcription step with random primers, PCR was performed using primers NVP3-Fdeg, NVP3-R1 and probe NVP3-Probe. The reaction mixture consisted of 1 × TaqMan Universal master mix (Invitrogen), 0.2 μM each primer, 0.15 μM probe and 0.05 μl ROX dye. Amplification conditions were two minutes at 50 °C and one minute at 95 °C, followed by 45 cycles of 15 s at 95 °C and one minute at 60 °C.

### Electron microscopy

Clinical material for electron microscopy was selected based on: (i) the rapid test method and positive result; (ii) PHE reference laboratory confirmatory PCR result; (iii) availability of material; (iv) most recent specimen to minimise sample degradation. Specimens (19 samples) screened by RORT2 (n = 4), RORT3 (n = 5), RORT4 (n = 5) or RART11 (n = 5) were included. Two positive samples and at least two negatives for the PHE reference laboratory confirmatory PCR per rapid test group were tested. Detailed methods are provided in Supplementary Materials and methods.

### Statistical analysis

Data analysis was performed by calendar year based on sample receipt date at the PHE reference laboratory. Statistical analysis was performed using MS Excel and Stata v14.1 software (StataCorp, Texas, US). Confirmation rates or positive predictive values (PPV), standard errors (SE) and 95% confidence intervals (95% CI) were calculated for all methods. PPV is the probability that the individuals with a positive screening result will truly have the disease. To test if there were any significant differences (p < 0.001) in PPV between the different testing methodologies and individual tests, chi-squared tests were performed.

### Ethical statement

PHE has legal permission, provided by Regulation 3 of The Health Service (Control of Patient Information) Regulations 2002 [14], to process patient confidential information for national surveillance of communicable diseases and includes PHE’s responsibility to monitor the safety and effectiveness of vaccines.

## Results

### Rotavirus confirmed-positive samples

During the two-year surveillance period, 3,729 RV samples that tested positive at local NHS hospital laboratories were received by PHE reference laboratory for confirmation and additional characterisation. Of those 3,729 samples, 3,721 were included in this data analysis based on the completeness of information required and the statistical value. Eight samples were excluded from this study because they did not meet the criteria: seven samples lacked information on the screening methodology used by the local NHS hospital laboratories and one sample had been tested using a commercial PCR test, putting it in a PCR4 category of n = 1.

All samples were subjected to the PHE reference laboratory confirmatory PCR test targeting VP6 gene before attempting strain characterisation. The PHE reference laboratory confirmatory PCR test was positive in 73% (2,716/3,721) of referred samples (Table).

**Table:** Rotavirus referred positive samples and confirmation rates, England, 2016–2017 (n = 3,721)

Test	Samples		Confirmed		SE	95% CI	
	n	Use (%)	n	PPV (%)		Lower	Upper
RORT 1	34	0.9	5	14.7	6.2	6.2	31.1
RORT 2	187	5.0	166	88.8	2.3	83.4	92.6
RORT 3	1,165	31.3	774	66.4	1.4	63.7	69.1
RORT 4	117	3.1	69	59.0	4.6	49.8	67.5
RORT 5	6	0.2	2	33.3	21.1	7.2	76.3
RORT 6	26	0.7	9	34.6	9.5	18.8	54.7
RART 7	5	0.1	4	80.0	20	25.6	97.9
RART 8	111	3.0	101	91.0	2.7	84	95.1
RART 9	48	1.3	43	89.6	4.5	77.1	95.6
RART 10	56	1.5	44	78.6	5.5	65.8	87.5
RART 11	244	6.6	189	77.5	2.7	71.8	82.3
RART 12	130	3.5	98	75.4	3.8	67.2	82.1
RART 13	16	0.4	12	75.0	11.2	48.2	90.6
RART 14	14	0.4	12	85.7	9.7	55.9	96.6
RART 15	29	0.8	21	72.4	8.4	53.4	85.7
RART 16	115	3.1	65	56.5	4.6	47.3	65.3
RART 17	305	8.2	132	43.3	2.8	37.8	48.9
EIA Commercial 1	248	6.7	230	92.7	1.7	88.8	95.4
EIA Commercial 2	233	6.3	199	85.4	2.3	80.3	89.4
EIA Other	97	2.6	76	78.4	4.2	69	85.5
PCR Commercial 1	80	2.1	37	46.3	5.6	35.6	57.3
PCR Commercial 2	44	1.2	27	61.4	7.4	46.2	74.6
PCR Commercial 3	6	0.2	6	100.0	0	NA	NA
PCR In-house	405	10.9	395	97.5	0.8	95.5	98.7
Total	3,721	100	2,716	73.0	NA	NA	NA

CI: confidence interval; EIA: enzyme immunoassay; NA: not applicable; PPV: positive predictive value; RART: rotavirus and adenovirus rapid test; RORT: rotavirus only rapid test; RV: rotavirus; SE: standard error.

### Use of primary screening tests

For the purpose of this analysis, local testing methods were grouped in three categories: immunochromatography-based or rapid test, enzyme immunoassays (EIA) and PCR tests. Most samples (2,608/3,721, 70.1%) had been tested with rapid tests locally, while 15.5% (578/3,721) had been tested by EIA and 14.4% (535/3,721) by PCR.

RORT3 was the most commonly used test with 1,165 (31.3%) samples followed by in-house PCRs (405 samples, 10.9%). RART17 (305, 8.2%), commercial EIA1 (248, 6.7%), RART11 (244, 6.6%) and commercial EIA2 (233, 6.3%) were less frequently used, while RORT5 (6, 0.2%), RART7 (5, 0.1%), RART13 (16, 0.4%), RART14 (14, 0.4%) and commercial PCR3 categories (6, 0.2%) were each represented with less than 20 samples (Table).

### PHE reference laboratory confirmation rates and positive predictive value

**Figure 1:**
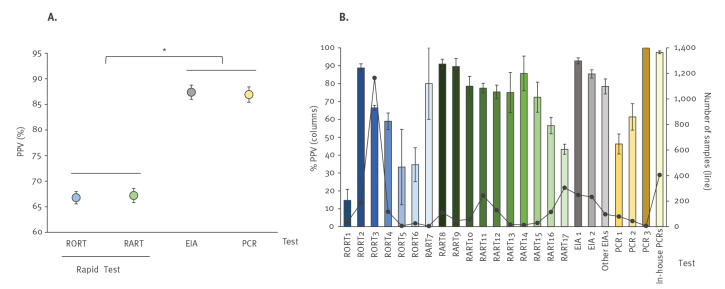
Positive predictive values of (A) rapid tests vs EIA and PCR and (B) individual testing method for rotavirus infection, England, 2016–2017 (n = 3,721)

EIA: enzyme immunoassay; PPV: positive predictive values; RART: rotavirus and adenovirus rapid test; RORT: rotavirus only rapid test; *: significant difference (p < 0.001).

Of the 2,608 specimens with a positive rapid test result, the PHE reference laboratory confirmatory PCR test was positive in 1,746 (66.9%) of cases. Analysis of the positive predictive value (PPV) by testing method showed a clear difference between the rapid tests when compared with the EIA and PCR methods (Figure 1A). PPVs were significantly higher (p < 0.001) for PCR (86.8%) and EIA (87.4%) methods compared with the RORT (66.8%) and RART (67.1%) rapid test methods. PPVs for individual tests ranged from 14.7% (5/34) for RORT1 to 91% (101/111) for RART8 (Table and Figure 1B). RV RNA was detected in less than 50% of samples initially tested with RORT1, RORT5, RORT6 or RART17. For the most commonly used rapid test, RORT3 (n = 1,165 specimens), RV RNA was confirmed in only 774 (66.4%) samples.

Within the EIA group, results were more consistent and less variable, with PHE reference laboratory confirmation rates ranging from 78.4% (76/97) for the other EIAs group to 92.7% (230/248) and 85.4% (199/233) for the two commercial EIA tests (EIA1 and EIA2, respectively).

Of the commercial PCRs used by local NHS hospital laboratories, there was variable and suboptimal performance overall, with PHE confirmation of 61.4% (27/44) for commercial PCR2 and 46.3% (37/80) for commercial PCR1. By contrast, 97.5% (395/405) of in-house PCR and six of six commercial PCR3 assays were confirmed as positive by the PHE reference laboratory PCR test.

### Seasonal variation

**Figure 2:**
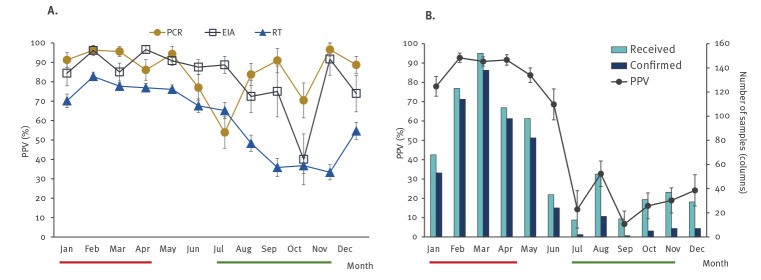
Positive predictive values by month and (A) testing methods and (B) rotavirus only rapid test, for rotavirus infection and month, England, 2016–2017 (n = 3,721)

EIA: enzyme immunoassay; PPV: positive predictive value; RORT: rotavirus only rapid test; RT: rapid test.

^a^ Most commonly used test, data for 2017 only. High and low RV seasons are indicated by red and green lines, respectively.

In order to assess whether the seasonal nature of RV activity may impact on PPV of screening tests, an analysis of the performance by methodology was conducted across each month. The rate of samples confirmed positive showed inconsistent variation per month (Figure 2A and Table S1). All methodologies showed a decrease in confirmation rates during low-season months (July to November) with lowest PPV for PCR occurring in July (21/39, 53.8%, 95% CI: 38.1%–68.9), for EIA in October (6/15, 40%, 95%CI: 18.6%–66.1) and for rapid tests in November (50/150, 33.3%, 95% CI: 26.2%–41.3). The PPVs of the rapid tests were the lowest of all methodologies for both high (January to April) and low seasons.

Analysis of seasonal variation on confirmation rates for RORT3, the rapid test with the highest number of specimens, showed a similar pattern but with greater variation (Figure 2B and Table S2). The greatest proportion of samples confirmed as positive was observed in February (114/123, 92.7%), with 89.6% (403/450) for the complete peak RV season (January to April). Outside the RV season, the confirmation rate was only 21.4% (32/149) with the lowest percentage observed during September (1/15, 6.7%).

### Detection of rotavirus NSP3 gene and electron microscopy

To further confirm our findings, selected samples were subjected to two additional detection assays. A subset of 59 specimens initially screened positive by rapid tests but negative by the PHE reference laboratory confirmatory PCR test, was tested by PCR amplification of the NSP3 gene. Only two samples (2/59, 3.4%) were positive indicating that 96.6% of the samples were true negatives. Cycle threshold (Ct) values for NSP3 tests for the two samples (Ct: 34.9 and 37.0) suggested the presence of RV at a very low genome content.

Electron microscopy (EM) was also performed on 19 samples to visualise any viral particles. Eight samples found positive by both rapid test and PHE reference laboratory confirmatory PCR test were all confirmed as positive by virus particle visualisation (data not shown), while eleven specimens with a negative PHE reference laboratory confirmatory PCR test result also failed particle detection under EM (Table S3).

## Discussion

Our results highlight the importance of validating screening results for RV diagnosis. The strong reproducibility of the results for referred samples that were screened by in-house PCR tests supports this methodology for RV detection. However, only 10.9% (405/3,721) of the samples were screened using this methodology. The commercial PCR tests PCR1 and PCR2 performed less well in comparison to in-house PCRs (confirmation rate of 46.3% and 61.4% for PCR1 and PCR2, respectively, compared with 97.5% for the in-house PCR). In addition, a greater proportion of PCR1 and PCR2 samples failed to be confirmed as positive at the PHE reference laboratory: 53.8% (43/80) of PCR1; 38.6% (17/44) of PCR2 and 2.5% (10/405) of in-house PCR samples failed. Commercial PCR detection kits are marketed as very specific and sensitive tests for RV detection but remarkably, the commercial PCR tests included in our analysis revealed a variable confirmation rate. It is unclear why in-house PCR assays out-performed commercial PCR assays. One possible explanation may be differences in assay validation, since the development of in-house assays could include more stringent local validation steps as part of the assay development process. Although the number of samples in the PCR category was relatively low (535/3,721, 14.4%), our results suggest that additional local verification of commercial tests may be required before routine use.

Performance of assays within the EIA group was very high and consistent overall. This finding is supported by previous reports highlighting the suitability of this testing method for surveillance programmes [15]. All three EIA groups in this analysis, consisting of both commercial and in-house assays, had very high confirmation rates. However, only a relatively small proportion (578/3,721, 15.5%) of total NHS hospital laboratory samples was tested using EIA.

Most NHS hospital laboratories prefer to use rapid tests for RV screening, rather than alternative methodologies. Cost and resources are likely to play an important part in this decision, since rapid tests are relatively inexpensive and easy to use compared with EIA and PCR assays that require dedicated equipment and trained staff. In contrast, rapid tests for RV are designed as point-of-care tests (POCT), which allow fast screening for rapid diagnosis, need no specialised equipment and can be performed by personnel with minimal or no specific laboratory training. In general, benefits of a POC testing approach are clear in terms of rapid administration of rehydration therapy, isolation or no admission into particular settings (i.e. hospital wards) and reduction in the use of ineffective treatments such as antibiotics. However, POCT for RV detection have limitations. Our analysis indicates that this popular screening method performs poorly, with only 66.9% (1,746/2,608) of locally positive samples being confirmed as positive by PHE. The overall PPV for IC tests in this study is also lower than recent reports [15-18]. Similar discrepancies for screening tests have been described in the literature. A high proportion of false positive results in Australia was reported after an unexplained surge in RV cases [19], and an excessive number of false positive results by IC tests was also reported in Spain [20].

To confirm our findings and support the PHE RV confirmation strategy, additional tests were performed on positive rapid test samples that failed PHE reference laboratory confirmatory PCR test detection. The rationale for this approach was to use two independent assays to confirm VP6 negative samples as true negatives. Results for the NSP3 detection confirmed that of the 59 samples available for retesting (with negative results by VP6 detection test), 57 specimens were true negatives, and two samples failed VP6 detection because of very low RV nucleic acid content. The second methodology undertaken was EM, which has conventionally been used as a reference method for RV detection [21]. The results of both these additional assays support the VP6 detection strategy as appropriate for RV confirmation. The limitations of these results are the low number of samples available or included for testing and the possibility of degradation of samples due to the time between the initial PHE reference laboratory confirmatory PCR tests and later NSP3 PCR and EM tests.

Our results suggest that out-of-season false positive results are more common for screening tests compared with tests performed during the RV season, particularly when rapid tests are used. For the most commonly used rapid test, RORT3, the confirmation rate was 92.7% (114/123) in February 2017, but only 1 in 15 in September 2017. A lower PPV can be expected during months when RV activity decreases and this will have an impact on PHE confirmation rates regardless of the screening methodology used by the NHS hospital laboratories. The higher proportion of false positive rapid test results when RV activity is lower will also have considerable implications for national RV surveillance because RV infections have fallen dramatically since the introduction of the infant immunisation programme [4,5]. The high rates of false positives, especially out-of-season, will underestimate the true impact and effectiveness of the current immunisation programme. More importantly, false positive results could have important implications for patient diagnosis and subsequent clinical management, and may divert efforts to investigate and identify the true cause of illness, which could delay the administration of appropriate treatment.

There are also cost implications in processing referred samples for molecular characterisation if a large proportion of samples are false positive. At the PHE reference laboratory, characterisation and typing is based on analysis and sequencing of RV VP4 and VP7 gene amplicons, which are both labour-intensive and resource-intensive assays.

The strength of this study lies in the availability of a single national reference centre for processing RV samples across England for the purpose of national surveillance. Consistent surveillance has been in place since the infant RV immunisation programme began more than 5 years ago, and large numbers of samples are processed using the same reference laboratory protocol every year. A limitation is the different number of samples analysed within the different categories, which limits the statistical power for tests with relatively small numbers of samples submitted to PHE. Our data, however, include results from every sample submitted from patients across England over a two-year period and, therefore, represents the state of RV testing at local and national level during 2016 and 2017. Another limitation is that there may be several reasons why local testing may be positive for RV but negative with the PHE reference laboratory confirmatory PCR test, such as small sample volumes, low RV concentrations or sample degradation. This, however, is likely to represent only a small proportion of the PHE confirmed negative samples, as supported by the results of the two additional detection assays performed on a subset of PHE confirmed positive and confirmed negative samples.

### Conclusion

A review of the methodologies used for RV initial detection showed a clear preference for rapid tests among NHS hospital laboratories. Rapid tests can be highly unreliable if used as the sole diagnostic method; even the best performing assays should be considered for screening only and should be confirmed using a more reliable, confirmatory test. Inconsistencies in confirmation rates for IC and other commercial assays, such as commercial PCRs, demonstrate the importance of a verification process before implementation into clinical settings. Furthermore, this report emphasises the need for a confirmatory result to support all screening tests for diagnosis of RV because the results may have implications for both the clinical management of patients and national surveillance. A reactive RV detection result using screening tests should be interpreted with caution if used to direct clinical management. Surveillance programmes monitoring the effectiveness of RV immunisation should be aware of high false positive rates with commonly used RV screening tests since they may underestimate vaccine effectiveness if reference laboratory confirmation rates are not considered alongside.

## Supplementary Data

818000 Supplement
