# Supplementary material for: Retrospective analysis on confirmation rates for referred positive rotavirus samples in England, 2016 to 2017: implications for diagnosis and surveillance
Source: Euro Surveill. 2020 Oct 29;25(43):1900375. doi: 10.2807/1560-7917.ES.2020.25.43.1900375 (PMC7596921; doi:10.2807/1560-7917.ES.2020.25.43.1900375)
Supplement: Supplement [file Supplement_1900375_DUNNING.pdf]

Supplementary material

This supplementary material is hosted by *Eurosurveillance* as supporting information alongside the article 'Retrospective analysis on confirmation rates for referred positive rotavirus samples in England 2016 to 2017: implications for diagnosis and surveillance', on behalf of the authors, who remain responsible for the accuracy and appropriateness of the content. The same standards for ethics, copyright, attributions and permissions as for the article apply. Supplements are not edited by *Eurosurveillance* and the journal is not responsible for the maintenance of any links or email addresses provided therein.

Table S1. Seasonal variation. Monthly number of samples, confirmed positives by VP6 detection assay, positive predictive values (PPV), standard error (SE) and confidence intervals (95%CI) were calculated for each group. Highest (*) and lowest (**) PPV values for each category are indicated.

	Rapid Test						EIA						PCR					
Month	Samples (n)	Confirmed (n)	Confirmed PPV (%)	SE	Lower CI	Upper CI	Samples (n)	Confirmed (n)	Confirmed PPV (%)	SE	Lower CI	Upper CI	Samples (n)	Confirmed (n)	Confirmed PPV (%)	SE	Lower CI	Upper CI
Jan	171	120	70.2	3.5	62.9	76.6	32	27	84.4	6.5	67.1	93.4	57	52	91.2	3.8	80.4	96.3
Feb	301	249	82.7*	2.2	78	86.6	51	49	96.1	2.7	85.4	99	80	77	96.3*	2.1	88.9	98.8
Mar	435	338	77.7	2	73.5	81.4	60	51	85.0	4.6	73.5	92.1	68	65	95.6	2.5	87.1	98.6
Apr	373	287	76.9	2.2	72.4	80.9	88	85	96.6*	1.9	89.9	98.9	43	37	86.0	5.3	72	93.7
May	349	266	76.2	2.3	71.5	80.4	131	119	90.8	2.5	84.5	94.7	35	33	94.3	4	79.4	98.6
Jun	179	121	67.6	3.5	60.4	74.1	72	63	87.5	3.9	77.6	93.4	39	30	76.9	6.8	61	87.7
Jul	133	86	64.7	4.2	56.1	72.3	53	47	88.7	4.4	76.8	94.9	39	21	53.8**	8.1	38.1	68.9
Aug	145	70	48.3	4.2	40.2	56.4	29	21	72.4	8.4	53.4	85.8	43	36	83.7	5.7	69.4	92.1
Sep	106	38	35.8	4.7	27.3	45.4	12	9	75.0	13.1	43.3	92.2	22	20	90.9	6.3	69.2	97.8
Oct	136	50	36.8	4.1	29.1	45.2	15	6	40.0**	13.1	18.6	66.1	27	19	70.4	9	50.5	84.7
Nov	150	50	33.3**	3.9	26.2	41.3	12	11	91.7	8.3	56.3	98.9	29	28	96.6	3.4	78.5	99.5
Dec	130	71	54.6	4.4	46	63	23	17	73.9	9.4	52.2	88	53	47	88.7	4.4	76.8	94.9

Table S2. Seasonal variation for rapid test RORT3 in 2017. Monthly number of samples, confirmed positives by VP6 detection assay, positive predictive values (PPV), standard error (SE) and confidence intervals (95%CI) were calculated for each group. Highest (*) and lowest (**) PPV values are indicated.

Month	Samples (n)	Confirmed		SE	Lower	Upper
		(n)	PPV (%)		CI	CI
Jan	68	53	77.9	5.1	66.5	86.3
Feb	123	114	92.7*	2.4	86.5	96.2
Mar	152	138	90.8	2.4	85	94.5
Apr	107	98	91.6	2.7	84.6	95.6
May	98	82	83.7	3.8	74.9	89.8
Jun	35	24	68.6	8	51.4	81.8
Jul	14	2	14.3	9.7	3.4	44.1
Aug	52	17	32.7	6.6	21.3	46.6
Sep	15	1	6.7**	6.7	0.9	36.9
Oct	31	5	16.1	6.7	6.8	33.8
Nov	37	7	18.9	6.5	9.2	35
Dec	29	7	24.1	8.1	11.8	43.1

Supplementary Materials and Methods

Electron microscopy

A 10% w/v faecal suspension was made in water for each sample. This was mixed thoroughly and allowed to settle overnight at +4C. The less turbid material at the top of the suspension was used for negative staining. 5µl of sample was incubated for 1 minute on a glow-discharged, carbon-pioloform coated 600 mesh thin bar electron microscopy (EM) grid. Excess sample was removed by blotting and grid surface washed twice with water, before being negatively stained for 10-15 seconds with 1.5% phosphotungstic acid (pH 6.5). The stain was removed to dryness by blotting and the grids were observed in a JEM1400 transmission electron microscope fitted with an AMT digital camera. For a semi-quantitative comparison, 25 grid squares were screened at 8000x mag for each grid and the number of rotavirus particles seen recorded.

Table S3. Particle visualisation by electron microscopy.

Sample ID	Method Referring Lab	Screening Result	VP6 qRT-PCR Result	Number of particles*
#1	RART11	Positive	Negative	0
#2				0
#3				0
#4			Positive	127
#5				1054
#6	RORT2	Positive	Negative	0
#7				0
#8			Positive	2315
#9				243
#10	RORT3	Positive	Negative	0
#11				0
#12				0
#13			Positive	117
#14				303
#15	RORT4	Positive	Negative	0
#16				0
#17				0
#18			Positive	30
#19				449

* Number of particles in 25 grids squares.
